# EHMTI-0168. Primary headache associated with sexual activity: fifteen new cases and therapeutic results

**DOI:** 10.1186/1129-2377-15-S1-D77

**Published:** 2014-09-18

**Authors:** K Zhu, Y Huang, J Chen

**Affiliations:** 1 Neurology, Panyu Hospital of Chinese Medicine, Guangzhou, China; 2 Neurology, Guangdong No.2 provincial people’s hospital, Guangzhou, China

## Introduction

Primary headache associated with sexual activity (PHASA) is a rare benign headache disorder.To our knowledge, this is the first Chinese mainland fifteen cases.

## Aims

To explore the clinical manifestations,diagnosis,treatment and prognostic features of PHASA.

## Methods

Fifteen patients,11 men,4 women,from 26 to 56 years old(mean 42±11),were prospectively analyzed over the past more than 7 years in our hospitals.The study was approved by our hospital Review Boardand and all of subjects have signed written informed consent.

## Results

Their ages of onset were from 26 to 44 years old(mean 38 ± 7 ). For preemptive therapy, 8 patients had received indomethacin (25–50 mg, intake 30–60 min prior to sexual activity). 7 patients reported good results and one limited success of this treatment and 4 patients had received sumatriptan (50-100mg, intake 30–60 min prior to sexual activity). 2 patients reported good results and remaining 2 had no response. They received ibuprofen for preemptive therapy complete success. Prophylactic treatment with propranolol was given in 8 patients. The dose was 60 mg for propranolol. Six patients reported good results, two no success of propranolol prophylaxis. Other drug applied for prophylaxis was nimodipine in 4 patients,two success and two without success.

## Conclusions

The patient who is diagnosed with PHASA should firstly receive indomethacin, or other drugs, sumatriptan, ibuprofen, is used for preemptive therapy if she/he can not get relief and(or)tolerate the adverse effects and should firstly receive propranolol, or other drug, nimodipine, is used for prophylaxis if she/he can not get relief and(or)tolerate the adverse effects.

No conflict of interest.

